# Current News and Opinion

**Published:** 1883-05

**Authors:** 


### Current News and Opinion.

### SOUTHERN DENTAL ASSOCIATION, AND GEORGIA STATE DENTAL SOCIETY.

Having received a special appeal from the South Carolina Dental Association, endorsed by prominent gentlemen in our profession from various parts of the United States, and approved by the Executive Committee of the Southern Dental Association, I do hereby direct, and it is so ordered, that the next meeting of the Southern Dental Association be held in Atlanta, Ga., commencing Tuesday, July 31st, 1883. This change is made to accommodate those in the South who wish to attend the Southern, and then go to the American Dental Association at Niagara Falls, afterward remaining North for their vacation. Also for those in our profession who belong to the order of Knights Templar, and are contemplating a pleasure trip to California, in August. The Georgia State Dental Society will meet at the same place, on Monday, July 30th. Respectfully Yours,

L. D. CARPENTER,
President Southern Dental Association.
D. HOPPS,
President Georgia State Dental Society.

ATLANTA, Ga., April 28th, 1883.

### THE MISSOURI DENTAL JOURNAL.

This sterling periodical too, is undergoing material changes. For the past five years it has been ably conducted as an independent journal, by Dr. C. W. Spalding, of St. Louis. But fresh enterprizes have engaged his attention, and he can no longer devote any time to professional journalism. He has therefore disposed of his interest, and henceforth the *Missouri Dental Journal* will be published from the Kansas City Dental Depot. Its editor is not yet announced, but it always was ably conducted, and we doubt not it will continue to be. While we regret the passing, we hail the coming management.

### UNION MEETING AT SPRINGFIELD, MASS.

The Massachusetts Dental Society, and the Connecticut Valley Dental Society, will hold a joint convention at Springfield, Mass., June 6th, 7th and 8th, prox. It is proposed to make this meeting one of the best ever held in New England. The executive committees of the two societies are actively engaged in completing arrangements, and have announced papers from the following gentlemen :—

Dr. W. G. A. Bonwill, Philadelphia;
Dr. R. R. Andrews, Cambridge;
Dr. T. H. Chandler, Boston;
Dr. D. M. Clapp, Boston;
Prof. Chas. Mayr, Springfield;
Dr. W. C. Barrett, Buffalo.

The setting of artificial crowns will be demonstrated in clinics by Dr. H. W. F. Buttner, of New York; Dr. H. A. Baker, of Boston; and Dr. E. P. Brown, of Long Island.

All dentists are cordially invited to attend the meeting, whether members of either society or not.

---

### COMPLIMENTARY.

Dr. J. Edward Line, Secretary of the Dental Society of the State of New York, won both gold and golden opinions at the late meeting. He was unanimously re-elected, and unanimously voted the sum of one hundred dollars—not in payment for, but in acknowledgement of his faithful and intelligent services. So much of the success of a dental meeting is dependent upon the secretary, that it is no wonder that when one like Dr. Line is found, every member is interested in keeping him in office as long as he can be persuaded to accept it.

